# In Vitro Inhibition of Replication of Dengue Virus Serotypes 1–4 by siRNAs Bound to Non-Toxic Liposomes

**DOI:** 10.3390/v14020339

**Published:** 2022-02-07

**Authors:** Carlos Andrés Rodriguez-Salazar, Delia Piedad Recalde-Reyes, Juan Pablo Bedoya, Leonardo Padilla-Sanabria, Jhon Carlos Castaño-Osorio, Maria Isabel Giraldo

**Affiliations:** 1 Center of Biomedical Research, Faculty of Health Sciences, Universidad del Quindío, Armenia 630003, Colombia; drecalde5552@cue.edu.co (D.P.R.-R.); jpbedoyaa@gmail.com (J.P.B.); lpadilla@uniquindio.edu.co (L.P.-S.); jhoncarlos@uniquindio.edu.co (J.C.C.-O.); 2 Molecular Biology and Virology Laboratory, Faculty of Medicine and Health Sciences, Corporación Universitaria Empresarial Alexander Von Humboldt, Armenia 630003, Colombia; 3 Department of Microbiology, Immunology University of Texas Medical Branch, Galveston, TX 77555, USA

**Keywords:** dengue virus, liposomes, siRNA

## Abstract

Dengue virus is a ssRNA+ flavivirus, which produces the dengue disease in humans. Currently, no specific treatment exists. siRNAs regulate gene expression and have been used systematically to silence viral genomes; however, they require controlled release. Liposomes show favorable results encapsulating siRNA for gene silencing. The objective herein was to design and evaluate in vitro siRNAs bound to liposomes that inhibit DENV replication. siRNAs were designed against DENV1–4 from conserved regions using siDirect2.0 and Web-BLOCK-iT™ RNAiDesigner; the initial in vitro evaluation was carried out through transfection into HepG2 cells. siRNA with silencing capacity was encapsulated in liposomes composed of D-Lin-MC3-DMA, DSPC, Chol. Cytotoxicity, hemolysis, pro-inflammatory cytokine release and antiviral activity were evaluated using plaque assay and RT-qPCR. A working concentration of siRNA was established at 40 nM. siRNA1, siRNA2, siRNA3.1, and siRNA4 were encapsulated in liposomes, and their siRNA delivery through liposomes led to a statistically significant decrease in viral titers, yielded no cytotoxicity or hemolysis and did not stimulate release of pro-inflammatory cytokines. Finally, liposomes were designed with siRNA against DENV, which proved to be safe in vitro.

## 1. Introduction

Dengue is a systemic viral infectious disease caused by the dengue virus (DENV), a flavivirus with a single-stranded positive polarity RNA genome of approximately 11 Kb, which is methylated on the 5′ end, and the 3′ end is not polyadenylated. It has a single open reading frame that encodes a single poly-protein, which is processed by cellular enzymes and viral proteases into 10 proteins; three structural (E: envelope, M: membrane-associated, and C: capsid) that form the viral particle and seven non-structural (NS1, 2A, 2B, 3, 4A, 4B, and NS5), which are necessary for processing and assembly of new viruses [1]. Currently, four serotypes are well established (named DENV 1–4 including various genotypes), all affecting humans [2].

This arbovirus is transmitted through the bite of female *Aedes aegypti* or *Aedes albopictus* mosquitoes. After the bite from an infected mosquito, the virus replicates in immature Langerhans cells; thereafter, these infected cells migrate to the local lymph nodes where the virus replicates and spreads in a hematogenous manner to various tissues including the liver, spleen, and bone marrow. Viral replication produces an infection with a broad spectrum of clinical presentations, ranging from subclinical or asymptomatic forms to severe forms of the disease characterized by abnormalities in hemostasis and increased vascular permeability, which may lead to hemorrhagic complications, organ damage, and even cause the death of the individual [3,4].

DENV infection is one of the most common arboviruses that affects humans, constituting a severe public health problem globally, especially in most tropical countries, where environmental conditions favor development and proliferation of *A. aegypti* and *A. albopictus*. Since 1995, it has been estimated that its distribution was comparable with malaria, and nearly 2.5-billion people lived in risk areas [5]. Currently, approximately 40% of the global population is at risk of DENV infection, with close to 390 million cases and increasing annually. In recent decades, greater reports of cases have been observed in almost all countries where the disease is found, with the population <15 years of age being the most affected [3,4,6,7]. A report by Ruiz et al., 2016 [8] registered the vector at altitudes of 2300 m, further broadening its distribution zone, reaching new populations susceptible to acquiring the disease [4,6,7,9].

Since 2006, the WHO has recommended research into antiviral molecules to treat dengue [10]; however, to date, there is no medication approved for specific treatment of this disease, and, although research has reportedly evaluated molecules that inhibit the virus entry into the cell, none of these have been used to treat patients. On the other hand, gene translation inhibitory molecules have been reported, such as small interfering RNAs (siRNA), double-stranded RNA molecules of approximately 21–25 mer, which have shown antiviral activity against various types of viruses during the last decade, including against flaviviruses, such as DENV [11,12,13,14,15,16,17,18,19,20].

These siRNAs inhibit expression of specific genes through the RNA-Induced-Silencing-Complex (RISC), through the antisense strand of the siRNA; this permits recognition of a messenger RNA (mRNA) by base complementarity for its degradation, which leads to the silencing of gene expression. It has been demonstrated in vitro and in vivo that these siRNAs can be administered exogenously and continue to carry out their role by joining RISC [16,21,22]. However, the correct delivery of these siRNAs to the cell has been challenging due to the poor stability of RNA in solutions, like plasma, or against immune and cellular system mechanisms. To solve this inconvenience, the use of liposomes or small lipid vesicles are a good alternative given their nature, which is similar to cell membranes, and they have already been used as systems of controlled release of different molecules [23,24,25,26,27,28].

Liposomes are vesicles composed of natural or synthetic phospholipids that form bilayers around an aqueous nucleus. These permit encapsulation of hydrophilic molecules in the aqueous interior and hydrophobic molecules within their bilayers. Liposomes are classified according to their morphology into: unilamellar (50–250 nm), multilamellar (500–5000 nm), or multivesicular and nano-liposomes (20–100 nm). It has been observed that large liposomes (50–500 nm) are phagocytosed and eliminated more quickly from the bloodstream [23]. Liposomes composed of neutral and cationic lipids that allow stable binding of nucleic acids, such as siRNA, have been growing in popularity lately given their capacity to interact preferentially with siRNA, their increased delivery efficiency, and tendency to be less toxic and more stable in the blood for uses in vivo [29,30,31]. This work sought to design lipid particles paired with siRNA capable of diminishing in vitro replication of the four serotypes of the dengue virus.

## 2. Materials and Methods

### 2.1. Cells and Viruses

Human hepatocarcinoma cell lines (HepG2) and baby hamster kidney (BHK) cells were obtained from the American Type Culture Collection (Manassas, VA). These were kept in culture medium (Eagle’s Minimum Essential Medium 1X (MEM) and 1X RPMI 1640 (Gibco by life technologies, Inc., USA), respectively, supplemented with 10% Fetal Bovine Serum (FBS) low in endotoxins and heat-inactivated (Life Technologies, Inc., Rockville, MD, USA) and 2 mM L-glutamine (Life Technologies, Inc., Rockville, MD, USA), 1X antibiotic (100 units/mL penicillin, 100 μg/mL Streptomycin) (Life Technologies, Inc., Rockville, MD, USA) and maintained at 37 °C with 5% CO_2_ atmosphere. DENV1–4 were isolated and cultured in C6/36 cells (kept in L15 medium supplemented with 10% tryptose (Life Technologies, Inc., Rockville, MD, USA) and 2% FBS), incubated for seven days at 28 °C and stored at −80 °C; the serotypes were verified through RT-Qpcr.

### 2.2. Obtaining Small Interfering RNAs

Initially, a search was made for complete genomes of each of the DENV serotypes through Pathogen Resource www.ViPRbrc.org. All the complete genome sequences were aligned with each other through Clustal Omega https://www.ebi.ac.uk/Tools/msa/clustalo/, accessed on 1 June 2021. Jalview (v.2.11.1.3), redundant sequences with >95% identity homology were eliminated, then conserved regions of the genome were selected to design siRNA through the siDirect 2.0 web server (http://siDirect2.RNAi.jp/, accessed on 1 April 2021) [32], adjusting parameters so the siRNA candidates complied with the Ui-Tei, Amarzguioui and Reynolds (URA) criteria to improve specificity in in vitro assays [33,34,35]. Additionally, consensus sequences for each serotype were used to generate siRNA candidates through the BLOCK-iT™ RNAi Designer web tool by ThermoFisher (https://rnaidesigner.thermofisher.com/rnaiexpress/, accessed on 1 April 2021).

Each siRNA candidate against DENV1–4 underwent a search through BLAST (Basic Local Alignment Search Tool) by NCBI to select specific siRNA against DENV genomes and avoid *off-target* sequences. Untranslated region (UTR) sites were avoided for each DENV serotype. Because the GC content of the siRNA duplex correlates with its functionality, a range from 30% to 52% was determined through OligoCalc http://biotools.nubic.northwestern.edu/OligoCalc.html, accessed on 1 April 2021 [36]. Structural and thermodynamic analysis was conducted using RNA structure (https://rna.urmc.rochester.edu/RNAstructure.html, accessed on 1 May 2021) to predict secondary structures in terms of their free bending energy at 37 °C, in addition to the interaction among the siRNA designed from the guide strand and its target as free binding energy, which is important for determining the effectiveness of the bond to the recognition sequence of each siRNA. Finally, the inhibition effectiveness of the siRNA was evaluated through the siRNApred web server (https://webs.iiitd.edu.in/raghava/sirnapred/, accessed on 1 May 2021).

The siRNA was synthesized by the Bioneer company Inc., Oakland, CA, USA as single-stranded oligos and as double-stranded oligos, reconstituted in ultrapure distilled water at 100 µM, and stored at −80 °C until use. In addition, the single-stranded oligos were prepared by using 1X alignment buffer (20 mM potassium acetate, 6 mM Hepes at pH 7.4, and 0.4 mM magnesium acetate) to generate double-stranded siRNA. Briefly, the protocol consisted of diluting each siRNA at a final concentration of 50 µM using water free of RNases. From these, 30 µL of each siRNA (sense and anti-sense) were combined to 50 uM plus 15 uL of 5X alignment buffer, obtaining a final volume of 75 uL. The final concentration of the siRNA duplex was 20 µM. This mix was incubated to form the duplex for 1 min at 90 °C and cooled slowly for 45 min until room temperature. Thereafter, the duplexes were incubated at 4 °C for 24 h and then stored at −80 °C until use.

### 2.3. Transfection of siRNA and Treatments with Liposomes-siRNA

An initial screening of the siRNAs was carried out via inverse transfection in HepG2 cells (4 × 10^5^ cells per well in six-well plates Nunc (ThermoFisher Scientific Rochester, NY, USA)), using RNAiMAX Lipofectamine (5 µL per well ThermoFisher Scientific in 250 µL OPTI-MEM Gibco for 20 min) at a final siRNA concentration of 40 nM (this screening was necessary to select which siRNAs would be used in treatments with liposomes-siRNA). The cells were kept with 2 mL/well of MEM maintenance culture medium (1X MEM, 2% FBS and 1% L-glutamine). The siRNA and lipofectamine mix were added to the culture of HepG2 cells in suspension, incubated at 37 °C in a 5% CO_2_ atmosphere for 12 h. Thereafter, the lipofectamine was eliminated and maintenance MEM medium was added and incubated at 37 °C with a 5% CO_2_ atmosphere for another 12 h.

Treatments with liposomes were carried out in HepG2 cells (4 × 10^5^ cells per well in six-well plates Nunc (ThermoFisher Scientific Rochester, NY, USA). For each well, 200 µL of liposomes were diluted in 200 µL of 1X OPTIMEM medium (Life Technologies, Inc., Rockville, MD, USA) for 20 min at room temperature, added to the culture of HepG2 cells and incubated at 37 °C with a 5% CO_2_ atmosphere for 12 h (the total volume was 2 mL and the final siRNA concentration was 40 nM). After 12 h, the medium was changed for maintenance MEM medium, incubated at 37 °C with 5% CO_2_ for another 12 h.

To determine the effect of the siRNAs on the dengue virus replication, the HepG2 cells transfected with siRNA (using lipofectamine or liposomes) were infected with DENV in 1X MEM culture medium without supplements for 2 h at 37 °C in 5% CO_2_ in independent assays with each of the serotypes at a multiplicity of infection (MOI) of 1. After the 2 h of infection, the cells were washed twice with 1X PBS, pH: 7.4 (130 mM NaCl, 3 mM KCl, 8 mM Na2HPO4 and 1.5 mM K2HPO4), and maintenance MEM medium was added followed by incubation for 30 h. After incubation, aliquots of the supernatant were made for subsequent analysis. The cells underwent total RNA extraction (through the Isolate 2 RNA mini kit from BioLine Ref. BIO52073 according to manufacturer’s instructions) to the evaluate viral gene expression through RT-qPCR. Finally, for each assay, cell viability was conducted through MTT (3-(4,5-Dimethylthiazol-2-yl)-2,5-Diphenyltetrazolium Bromide) or through cell staining by crystal violet. The effectiveness of the transfection into HepG2 cells was determined by using siRNA (FITC Conjugate)-A Santa Cruz Ref. Sc-36869, via fluorescence microscopy through the EVOS fl Cell Imaging System microscope (Thermo Scientific). Infected cells were used as positive control with each DENV serotype without treatments, siRNA-A (Santa Cruz Ref.: sc-37007) and cells without infecting and without treatment as negative control.

#### Evaluation of Cell Viability

Cell viability was evaluated to determine the siRNA concentration to be used in the antiviral assays. Likewise, post-treatment cell viability was evaluated to determine that the reduction in viral titers was due to action of the siRNAs and not due to decrease in the number of cells at the end of each assay. Cell viability evaluation was conducted through two techniques; one that determines cell metabolism through the MTT technique, according to the manufacturer, and another through staining viable cells adhered to the plate through crystal violet [37].

### 2.4. Plaque Assay

BHK cells/well were seeded in 24-well plates, in RPMI 1640 maintenance medium (1X RPMI 1640, 2% FBS, 1X antibiotic, 1% L-glutamine) and kept at 37 °C with 5% CO_2_ for 24 h. Then, the cells were washed twice with 1X PBS to remove the maintenance medium, adding 180 µL of 1X RPMI 1640 plus 20 µL of the virus (supernatants of treatments, infection controls, and cell controls). From this mixture, five serial dilutions were carried out, then the infection process was incubated at 37 °C with 5% CO_2_ for 2 h, discarding the inoculum and adding 1 mL of the overlay medium (2X RPMI 1640, medium viscosity carboxymethylcellulose at 1.2% (final concentration), 2% FBS, 1X antifungal antibiotic, 2% sodium bicarbonate) and incubated at 37 °C with 5% CO_2_ for seven days. Finally, the cells were fixed with 4% formaldehyde in 1X PBS for 2 h at room temperature. The fixation solution was removed, and the cell monolayer was washed with abundant water. Staining medium was added (crystal violet (Sigma) at 2.5% diluted in 20% ethanol (Fisher) and 1X PBS) for 1 h at room temperature, excess stain was washed with tap water, and the viral titer was obtained through plate count.

### 2.5. RT-qPCR

After infection with DENV1–4, the HepG2 cells were collected and the genetic material was extracted through the Isolate 2 RNA mini kit by BioLine, following the protocol according to its manufacturer. The primers used were: DENV 7764 Fwd 5′-CGTCGAGAGAAATATGGTCACACC-3′, DENV 7844 Rev 5′-CCACAATAGTATGACCAGCCT-3′, (no siRNA in this study overlaps with the RT-qPCR primers used), hGAPDH Fwd 5′-TGTTGCCATCAATGACCCCTT-3′, hGAPDH Rev 5′-CTCCACGACGTACTCAGCG-3′, at 0.2 µM, which amplify a region of the NS5 protein [38]. The total RNA was transcribed to a complementary DNA (cDNA) through SuperScript™ III Reverse Transcriptase by ThermoFisher at 45 °C for 20 min and later amplified through the enzyme from New England Biolabs Luna Universal qPCR Master Mix according to manufacturer’s recommendation with 20 µL. The mix was standardized to a single step. A negative control without RNA template was included for all the RT-qPCR reactions, as well as a control with sample, but without RT and a control without infection.

### 2.6. Formulation and Characterization of Liposomes-siRNA against DENV 1–4

Liposomes were generated by using the thin layer hydration method. The organic solution of lipids consisted of the ionizable amino lipid dilinoleylmethyl-4-dimethylaminobutyrate (DLin-MC3-DMA) synthesized by MedKoo Biosciences, 1,2-distearoyl-sn-glycero-3-phosphocholine (18:0) (DSPC, Sigma), and cholesterol (Col, Sigma) with a molar ratio of 51:10:39, respectively, dissolved in chloroform and ethanol at a 3:1 ratio, the final lipid concentration was adjusted to 5 mg/mL. The organic solvents were then evaporated under pressure at −0.37 bar and 60 °C for approximately 2 h through the Heidolph rotavapor (Merck) to obtain a thin film. Finally, the balloon was covered with Crystaflex and stored at 4 °C overnight to permit total evaporation of the solvent.

Hydration of the thin layer was carried out using 2 mL of an siRNA solution (400 nM) in 1X PBS (Gibco) free from RNases (Gibco), at 37 °C upon obtaining multilamellar liposomes; size reduction was achieved through two sonication steps at 40 kHz for 1 min in a water bath at 37 °C, followed by extrusion steps through 0.22-µm and 0.1-µm filters (Santa Cruz). Finally, the liposome solution was incubated at room temperature for 30 min to permit its stabilization.

Size characterization was carried out through dynamic light scattering to determine liposome size through Zetasizer Nano ZS90 Malvern Panalytical^®^ to measure particle size and Z potential. Additionally, liposomes were observed by microscope at 100X and by fluorescence at 60X through staining with Hoechst (Bisbenzimide H 33258 Fluorochrome, Trihydrochloride, Sigma) pre- and post-extrusion.

The efficiency of encapsulation of siRNAs in liposomes was performed through 12% polyacrylamide gel electrophoresis for 90 min at 120 V. The study used siRNA without encapsulating and liposomes-siRNA; the intensity of the liposome band was compared with the unencapsulated duplex siRNA. The gels were visualized under ultraviolet light stained with 0.1% ethidium bromide and visualized in transilluminator filters 254/365 nm MUVB-122 Major Science. The photographs were then analyzed by using Fiji software [39].

Encapsulation effectiveness was determined through the following formula: Encapsulation effectiveness% = siRNA loaded to the liposome—siRNA not encapsulated/total siRNA loaded to the liposomes X100.

To determine the efficiency of the transfection, liposomes were prepared with siRNA (FITC Conjugate)-A Santa Cruz Ref. Sc-36869 and visualized through fluorescence microscopy through EVOS fl microscope Cell Imaging System (Thermo Scientific). Infected cells were used as a positive control for each DENV serotype without treatments. Negative controls were siRNA-A Santa Cruz Ref.: sc-37007 and cells without infecting and without treatment.

### 2.7. Hemolysis Assay of Human Erythrocytes

Through venous puncture, 3 mL of peripheral blood were taken in Vacutainer tubes with EDTA K3 (BD^®^), centrifuged at 400× *g*, for 5 min. The plasma and leukocyte layer were removed. The remaining red blood cells were washed four times using 4 mL of sterile 1X PBS. A total of 90 µL of erythrocytes prepared were deposited in 96-well plates plus liposomes with specific siRNA at a final concentration of 40 nM. The plate was incubated for 2 h at 37 °C, then centrifuged at 400× *g* for 5 min. The supernatants were transferred to a new 96-well box and read through absorbance at a length of 541 nm in the Epoch Biotek^®^ spectrophotometer. The percentage of hemoglobin released was calculated using the following formula: % hemolysis = (Absorbance of the sample–Absorbance of the negative control/Absorbance of positive control–Absorbance of negative control) × 100. 1X PBS was used as negative control and 0.01% Triton × 100 as positive control.

### 2.8. Dot-Blot Type Immunoassay to Evaluate Interleukin Release (IL-1β, TNF-α, IFN-γ and IL-6) from Whole Blood and Production of NS1 Protein Post-Infection in HepG2 Cells

Through venous puncture, 10 mL of peripheral blood were taken in Vacutainer tubes with sodium heparin (BD^®^ New Jersey USA); for each assay, 250 µL of whole blood diluted with 250 µL of 1X RPMI 1640 was used in 1.5 mL tubes and stimulated with each of the liposomes loaded with siRNA at 40 nM diluted in 1X RPMI 1640 to complete a final volume of 500 µL. This mix was incubated at 37 °C with 5% CO_2_ and 80% humidity for 8 h. After the incubation period, each tube was centrifuged at 400× *g* for 5 min. The supernatant from each assay was aliquoted and stored at −80 °C until evaluation by Dot-Blot. The protocol was based on Durhuus et al., 2021 [40]. As negative control, whole blood was used without stimulation. As positive control, lipopolysaccharide was used at a concentration of 100 ng/mL (Sigma Aldrich Cat. L4391-1mg).

Evaluation of the production levels of the NS1 viral protein was conducted from the supernatants post-infection, and treatments carried out with liposomes-siRNA in HepG2 cells. The Dot-Blot assay was performed by using Ultracruz^®^ PVDF Transfer Membrane, 0.45 µm (Santa Cruz, Dallas, Texas, USA), submerged for 20 min at room temperature in absolute methanol. Thereafter, 2 µL of each sample diluted in 1X PBS 1:500 were fixed on the membrane. The membrane was blocked at 4 °C overnight using 5% skim milk in 1X PBS-T (1X PBS and 0.05% Tween 20,). Anti-human antibodies were used, developed in mice (BioLegend IL-1b Ref. 508304, IL-1b Ref. 501204, IFN-gamma Cat. 502502, TNF-alpha Cat. 502802), and diluted in 1X PBS according to the manufacturer’s instructions. Analysis of the NS1 protein used a monoclonal antibody developed by the Molecular Immunology Group at Universidad del Quindío. The antibodies were incubated for 2 h at 37 °C; anti-mouse IgG conjugated with alkaline phosphatase was used as secondary antibody (Sigma Aldrich Ref. A4656), diluted in 1X PBS 1:10.000; incubated at 37 °C for 2 h. BCIP^®^/NBT Liquid Substrate System was used as substrate (Sigma Aldrich Cat. B1911 USA). The reaction was incubated for 30 min at 37 °C and was detained by washing with abundant water. A photographic record was made of the results and, subsequently, an intensity analysis was performed by pixel area using the Fiji software [39]. Between each incubation step, three washes were carried out by using 1X PBS-T (1X PBS plus 0.05% Tween 20).

### 2.9. Statistical Analysis

Three independent assays were carried out in triplicate for a single-factor analysis of variance to determine if differences exist among the groups: treated, positive controls, negative controls, and infection. A Shapiro–Wilk test was performed to determine normality in the data. Multiple comparisons tests were performed to determine among which groups statistically significant differences are occurring compared to the infection control using Tukey’s multiple comparison test. These tests were run through the Graphpad Prism v 9 statistical package (San Diego, CA, USA). All the tests conducted were considered statistically significant when *p* < 0.05 with 95%CI.

## 3. Results

### 3.1. Selection of Sequences and Obtaining siRNA

1845, 1419, 922, and 220 complete genomes were downloaded for DENV 1, 2, 3 and 4, respectively. Once redundant sequences were eliminated, (sequences with >95% identical) six different sequences were used for analysis for DENV1, 13 for DENV2, four for DENV3, and seven for DENV4. Subsequently, multiple alignments were performed through Clustal omega to obtain consensus sequences for each serotype. The consensus sequences were used to get siRNA candidates through the Invitrogen BLOCK-iT™ RNAi Designer platform using the Stealth RNAi™ siRNA option. Twenty different siRNA for a total of 100 siRNA candidates were obtained. Moreover, a total of 72, 30, 53, and 91 regions conserved for DENV1, DENV2, DENV3, and DENV4, respectively, were used in siDirect to predict siRNA. The guanine cytosine was between 36% and 48%, the possible bending options and binding energies ranged from −5.2 to 2.1 and the binding free energy was in a range from −48.4 to −31.7 with inhibition efficiencies of about 1, which makes it possible to predict its usefulness for in vitro evaluation. The recognition site of each siRNA in the viral genome was in regions that encode viral proteins (Table 1). Finally, after all the computational analyses, three siRNA per DENV serotype were synthesized.

### 3.2. Treatments with siRNA Show a Decrease in Viral Titers

**Figure 1:**
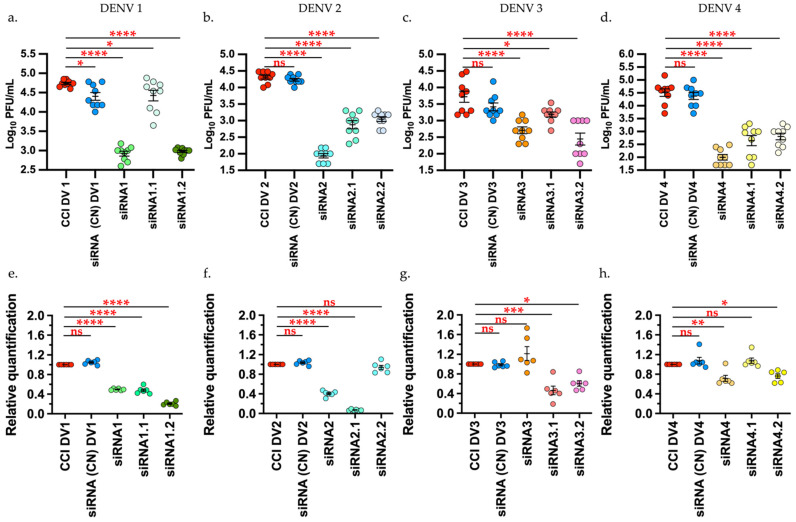
siRNA shows a decrease in the viral titers and relative quantification of viral genome of the DENV1–4 by plaque assay and RT-qPCR, respectively. Upper row. Effect of siRNA on the production of infectious viral particles (**a**) DENV1. (**b**) DENV2 (**c**) DENV3 (**d**) DENV4. Lower row. Expression level through relative quantification via RT-qPCR 2^−ΔΔ^Ct normalized by GAPDH. e. DENV1. f. DENV2 g. DENV3 h. DENV4. Infection control (CCI), Negative control (CN) Tukey’s multiple comparisons test * *p* < 0.05 ** *p* < 0.001 *** *p* < 0.0001 **** *p* < 0.00001. Graphic and analysis constructed in GraphPad-Prism 9.

The effect of siRNAs on the production of infectious viral particles on DENV 1 showed a significant decrease (*p* < 0.00001) of 98.4% and 98.2% for siRNA1 and siRNA1.2, respectively, compared with the infection control (CCI). For siRNA1.1, there was significant decrease (*p* < 0.05) by 51%. In comparison with the viral titer, the expression level through relative quantification (RT-qPCR) of the viral genes also displayed a decrease with a statistically significant difference (*p* < 0.00001) in viral copies of the three siRNAs evaluated (Figure 1a,e).

For DENV2, there was significant decrease (*p* < 0.00001) of the viral titers compared to the infection control (CCI) and negative control with 99.57%, 96.36%, and 94.6% for siRNA2, siRNA2.1, and siRNA 2.2, respectively. The expression level of viral genes through relative quantification for this virus revealed decreases with a statistically significant difference (*p* < 0.00001) only for siRNA2 and siRNA2.1, while for siRNA2.2, there was no difference against the infection control via RT-qPCR (Figure 1b,f).

The DENV3 results showed a significant decrease (*p* < 0.00001) with 90% reduction for siRNA3, 94.9% decrease for siRNA3.2 of viral titers, compared with the controls. For siRNA3.1, there was a significant decrease with *p* < 0.005 and 69.8% inhibition. The expression level of viral genes through relative quantification for this virus showed a significant reduction (*p* = 0.0005) for siRNA3.1 and (*p* = 0.0126) for siRNA3.2, while siRNA3, unlike plaque assay, did not show a significant difference against the infection control through RT-qPCR (Figure 1c,g).

In relation to the assays with DENV4, there was a significant decrease (*p <* 0.00001) with 98%, 91.2%, and 87.4% viral inhibition for siRNA4, siRNA4.1, and siRNA 4.2, respectively, compared to the controls. The expression level of viral genes through relative quantification for this virus showed significant decrease (*p* < 0.0063) only for siRNA4 and siRNA4.2 (*p =* 0.0281), while for siRNA4.1, unlike plaque assay, no difference existed against the infection control through RT-qPCR (Figure 1d,h).

After the transfection and infection, cell viability of the HepG2 cells was evaluated to determine that the variation of the viral titers was due to the effect of the siRNA and not because of cell integrity failures. According to this, it was evidenced that cell viability at the end of each assay remained around 100%. Moreover, the transfection effectiveness with lipofectamine was determined through siRNA (FITC Conjugate) A, where fluorescence was observed within the cell (Supplementary Figure S1).

Although the siRNA1, siRNA1.1, siRNA2, siRNA2.1, siRNA3.1, siRNA3.2, siRNA4, and siRNA4.2, showed antiviral activity, only siRNA1, siRNA2, siRNA3.1, and siRNA4 were evaluated in liposomes in this work.

### 3.3. The Liposomes-siRNA (L-siRNA) Structure and Concentration Does Not Affect Cell Viability, Not Produce Hemolysis and Stimulation of Interleukins (IL-1β. TNF-α. IFN-γ. and IL-6)

**Figure 2:**
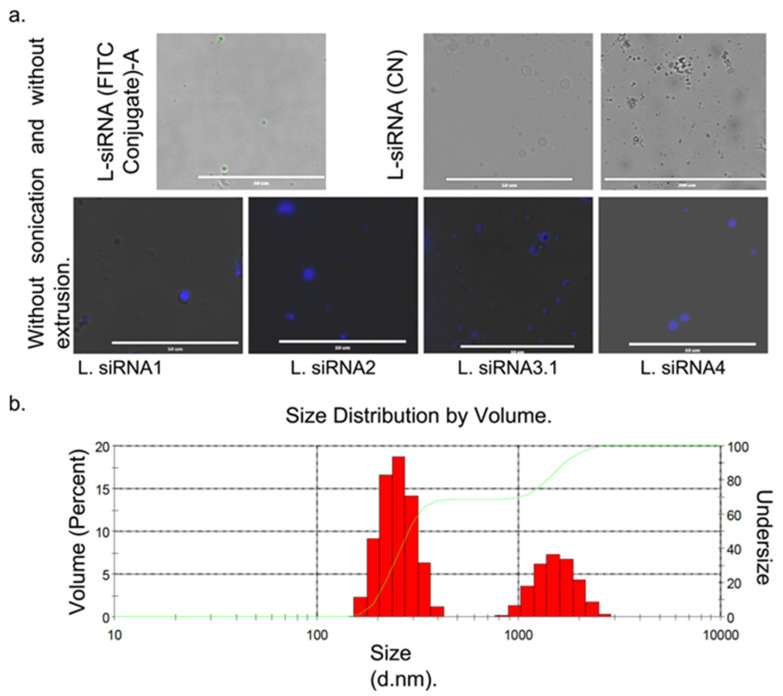
Characteristic sizes of liposomes with siRNA were obtained by fluorescent microscopy and dynamic light scattering (DLS). (**a**) liposomes with siRNA without reduction in multilamellar size. Upper left row liposomes fluorescing in green due to the L-siRNA (FITC Conjugate)-A. Right L-siRNA (CN). Lower row liposomes with siRNA against each DENV serotype treated with Hoechst. (**b**) The mean size distribution of the L-siRNA is given in diameter values in nanometers (d.nm) through DLS.

The final lipid concentration was adjusted at 5 mg/mL and 400 nM of siRNA. Before extrusion and sonication, multilamellar structures were observed. Once size reduction was made, it was not possible to observe structures under the fluorescence microscope, so they were evaluated through DLS, where they exhibit a bimodal behavior with a peak of 249.8 nm and 1535, possibly due to aggregates. The study obtained 75% encapsulation effectiveness for siRNA1; 80% for siRNA2; 68% for siRNA3.1, and 71% for siRNA4. Correct cell delivery was determined through fluorescence with siRNA (FITC Conjugate)-A with 73.5% encapsulation effectiveness, Figure 2a,b, respectively.

It was noted that the interaction of the erythrocytes with the four L-siRNAs did not generate hemoglobin release as a consequence of erythrocyte lysis and did not modify the morphology of the erythrocyte. Upon conducting each of the treatments with the L-siRNA and infection with each serotype, an evaluation was performed of the cell culture viability, finding that cell viability remained around 100%, which suggests that the reduction in viral titers was due to the treatments and not due to cell damage (Supplementary Figure S2).

**Figure 3:**
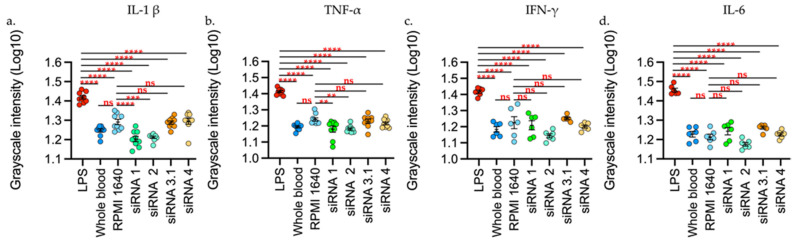
L-siRNAs did not increase the interleukins levels evaluated. (**a**) L-1β. (**b**) TNF-α. (**c**) IFN-γ. (**d**) IL-6. The stimuli are given by: LPS (Lipopolysaccharide 100 ng/mL), RPMI 1640, liposomes-siRNA (L-siRNA1, L-siRNA2, L-siRNA3.1, L-siRNA4. As negative control, blood was used without stimulus (blood). Data are expressed as Mean + SEM of the three independent assays in triplicate. The measurement is given by the intensity in grayscale in an equivalent area in all the samples (911 square pixels). ** *p* < 0.001 *** *p* < 0.0001 **** *p* < 0.00001. The measurement was performed with Fiji and was represented as Log_10_ through GraphPad Prism 9.

Ex-vivo analysis with whole blood showed that stimulation with LPS induced production of the cytokines evaluated with a statistically significant difference (*p* < 0.0001), while treatments with L-siRNA showed no statistically significant increase against the blood control or against the RPMI 1640 control (vehicle control) (Figure 3).

### 3.4. Liposomes-siRNA Inhibit the Viral Replication and Decrease NS1 Expression

**Figure 4:**
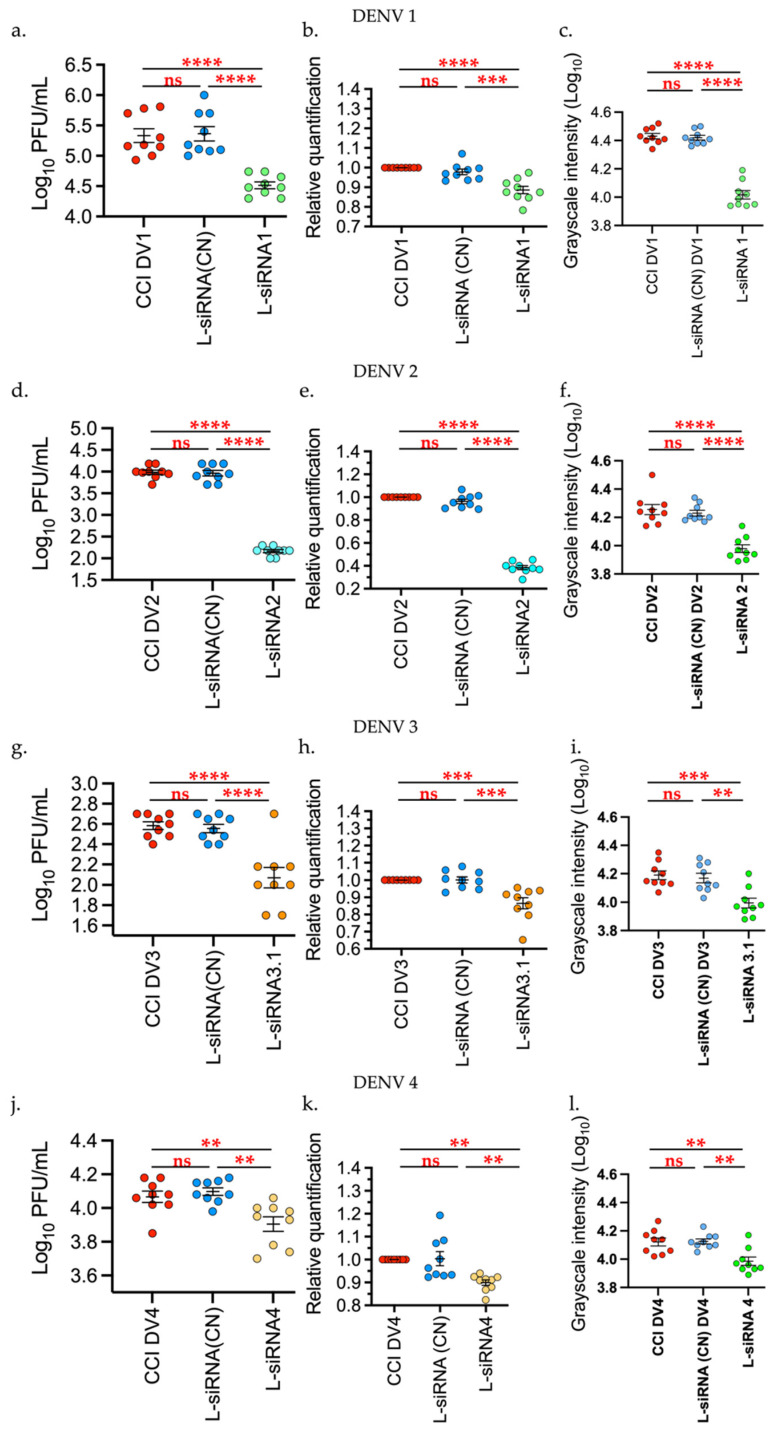
Liposomes-siRNAs inhibit the DENV 1–4 replication. The evaluation was performed through: (**a**,**d**,**g**,**l**) Plaque assay, (**b**,**e**,**h**,**k**) relative expression of viral RNA through RT-qPCR, and (**c**,**f**,**i**,**l**) production of the NS1 protein via dot blot; the last was measured through intensity in grayscale in an equivalent area in all the samples (911 square pixels), analysis was performed with Fiji and was represented as Log_10_. Infection control (CCI DV), Negative control (L-siRNA (CN)). Data are expressed as Mean + SEM of the three independent assays in triplicate. Tukey’s multiple comparisons test. ** *p* < 0.001 *** *p* < 0.0001 **** *p* < 0.00001. Graphics and analysis constructed through GraphPad Prism 9.

It was found that the effect of L-siRNA1 on the production of infectious viral particles on DENV1 had a statistically significant decrease (*p* < 0.00001) with 85.2% compared with the infection control (CCI DV1) and the negative control (L-siRNA (CN)). By comparing the expression level, measured through relative quantification (RT-qPCR) of the viral genes for DENV1, it was observed that the L-siRNA1 diminished the generation of viral copies with *p* < 0.0001 compared with the infection control and p < 0.0002 against the negative control. Additionally, production of the NS1 protein was also diminished significantly (*p* < 0.0001), Figure 4.

For DENV2, the decrease in infectious viral particles, viral genome, and NS1 production was evident, with *p* < 0.0001, and 98.4% inhibition against the infection control and negative control. With DENV3, the viral titer showed a significant reduction with *p* < 0.0001, and 69% inhibition in relation to controls, while the relative quantification of the viral genome had a *p*-value of 0.0004 in relation to the positive control and *p* = 0.0003 in relation to the negative control. The decrease in the NS1 protein was significant at *p* = 0.0001 against both controls.

With respect to DENV4, treatments with L-siRNA4 showed a statistically significant difference with *p* = 0.0071 and with 30% inhibition in relation to the infection control and *p* = 0.0015 in relation to the negative control, through plaque assay. The RT-qPCR data yielded a statistically significant decrease, *p* = 0.0032 in relation to the infection control and *p* = 0.0023 against the negative control. NS1 production was also significantly decreased with *p* = 0.0332 against the infection control and *p* = 0.0235 against the negative control (Figure 4).

## 4. Discussion

Dengue virus is known for being the etiological agent responsible for dengue disease. Currently, no antiviral agent or effective vaccine exists against the four serotypes, and the only licensed vaccine produced by Sanofi Pasteur (Dengvaxia**^®^**) has low effectiveness against serotypes 1 and 2 and may only be used to immunize individuals who have had a previous infection. On the contrary, infection with dengue virus in immunized individuals would provoke a serious reaction [41]. Thus, development of antiviral strategies is a necessary option to treat the disease, to diminish the viral load and reduce complication of clinical manifestations.

The study of molecules, like siRNAs, which recognize the virus genome directly, is an alternative to studying the use of drugs. These siRNAs naturally regulate mechanisms of gene expression within the cell, regulating expression of mRNA through RISC—a protein complex that recognizes messenger RNA (mRNA) through the 3′-5′ sense RNA strand of the siRNA, which is complementary with the mRNA. This interaction permits degradation of the mRNA or their repression [21]. Therefore, considering the characteristics of DENV replication, in which the single-stranded, positive-sense RNA genome behaves as a mRNA, it is possible to regulate viral expression through siRNA; however, their correct delivery within the cell is a challenge to consider for in vivo use [6,15,42,43].

To design an siRNA against the four DENV serotypes in this work, it was important to recognize the characteristics of the siRNA that relate to its interfering function and the characteristics of the viral replication. Another detail to bear in mind is the mutation rate of DENV, which varies between 103 and 105 nucleotides per replication round, where mutants can be generated in sequences that encode viral proteins essential for viral or structural replication. Such mistakes will generate inactive viral particles, and therefore, comparisons of infectious viral particles make apparent conserved regions that should be considered for designing siRNAs against this virus [6]. For this reason, only conserved sequences of the viral genome were considered for each serotype, which would be linked to sequences that generate functional products to obtain viral proteins. Although it has been shown that the untranslated regions (UTR’s) towards the 5′ and 3′ ends have highly conserved regions—given their importance in the replication process, they were not taken into account to obtain the siRNAs proposed in this work because these regions acquire secondary complex structures necessary for ribosomal recognition and their subsequent translation. It has been shown that the effectiveness of siRNAs depends on the accessibility of the mating zone on the viral RNA, and the presence of secondary structures on its target or binding of proteins in that area can prevent siRNA binding, reducing its effectiveness in silencing [6,21,44,45].

Candidate siRNAs obtained for evaluation against DENV in this work showed specific recognition over DENV sequences; sequences recognizing genes with functions in cell processes were not considered due to the possible risk of toxicity and were discarded from the in vitro evaluation process. Additionally, given that it has been shown that the GC content of the siRNA duplex correlates with its functionality, the siRNAs obtained in this work were selected by taking into account a percentage between 30% and 52% [34] (high GC content diminishes duplex unwinding rate and low GC content (<30%) can decrease hybridization and efficiency of target mRNA recognition). Furthermore, prediction of the secondary structures of the siRNA depends on the GC content and on the heat capacity (HC), thus, changes in the structures of the nucleic acids are not only given by enthalpy and entropy, but also by changes in their HC. When a double-stranded structure merges into single strands, its hydration changes and its heat capacity increases. Therefore, this depends on the melting temperature (Tm), which would be involved in the structural determination of the oligos and their concentration. Therefore, the thermodynamic characteristics were evaluated to determine the capacity of the sequences to generate structures and generate complementarity with their specific target through RISC [46,47]. Although the computational analyses already mentioned seek to generate siRNA sequences with favorable predictions for silencing in cell models, a functional result of these siRNAs is actually obtained only after in vitro or in vivo evaluation. This initial evaluation must overcome certain barriers to induce gene silencing. Given that siRNA have high molecular weights (13300 to 16000 for siRNA designed in this work), anionic charges, and are hydrophobic, they do not easily pass through the membrane, in addition to being targeted by nucleases [26,48]. Due to these factors, liposome-type lipid particles are an alternative for accurate and efficient delivery of siRNA designed through computational tools. The siRNA obtained in this work were evaluated initially in vitro, using lipofectamine, and efficiency in inhibiting expression of the viral genome was determined at a concentration of 40 nM. In accordance with the literature, the range in which the siRNAs have in vitro function is between 10 and 100 nM [49,50,51]. It has been observed that concentrations above these values may be cytotoxic, as noted in this study (data not shown), which exhibited diminished cell viability at concentrations >100 nM. In addition, Villegas et al., 2010 [52], managed to reduce viral titers by approximately one log. In the present work, the initial screening with lipofectamine showed a decrease of around two orders of magnitude (between 98% and 99% inhibition for siRNAs 1, 2, and 4) for Dengue 1, 2, and 4 and close to 69.8% for Dengue 3 (siRNA 3.1). It should also be highlighted that the evaluation through RT-qPCR revealed similar results against the plaque assay in some cases, except with siRNA 3, siRNA 4.1 (Figure 1), which may be because siRNA only knocks down gene expression, so the silencing is incomplete with some partial gene expression still occurring. According to Phei et al., 2020 [53], the amount of mRNA and siRNA influences the silencing success. In addition, fragments can be generated that cause false positives using certain techniques, like PCR or micro-arrangements. This gives strength to the idea of using the plaque assay as the main test to evaluate antiviral molecules.

Our results in in vitro evaluation with lipofectamine permits our preliminary determination of the function of our designed siRNA’s before conducting a preparation in liposomes for in vivo use. Evaluation with lipofectamine is only used in vitro because it has certain limitations, such as its transfections effectiveness varies by the types of cells used and siRNA + lipofectamine concentration, besides resulting toxic for use in live organisms. In this work, this process was standardized through inverse transfection in HepG2 cells, as a human cell model. Prior studies have shown the effectiveness of gene silencing of dengue virus genes and other viruses using this same cell model, although these have also been evaluated in BHK, MCCD, Vero cells, etc., with similar results; however, the delivery methods differed from those in the present study [15,25,52,54,55,56,57,58,59,60,61,62]. The formulation of liposomes for use as a transfection method provides certain advantages over lipofectamine, such as reduction in toxicity and immune stimulation. This particular liposome formulation and transfection method has been used during the last decade as a method of transferring nucleic acids for gene silencing or expression to use in vivo specially to treat cancer [15,25,52,54,55,56,57,58,59,60,61,62]. One of the objectives of conducting this work was to formulate a system that would permit evaluation in in vivo models. Therefore, a search was made for different strategies to provide the ideal siRNA delivery system within the cell. Authors, such as Cullis et al., have designed lipid nanoparticles for siRNA delivery to hepatic cells or cells of the immune system [24,26,48,63,64,65]. From these molecules, we highlight those using cationic lipids for potential use in in vivo therapy. These lipids have positive charges that interact with the phosphate groups of nucleic acids through electrostatic interactions, permitting generation of stable lipid particle-nucleic acid interactions and controlled siRNA release through the endosome within the cell [66].

The formulation of lipids to encapsulate siRNA was carried out through D-Lin-MC3-DMA, DSPC and Chol, with a molar ratio of 51:10:39. This formulation significantly reduces the viral titers in the four DENV serotypes in vitro (Figure 4). The viral titer showed a significant decrease of 85%, 98%, 69%, and 30% for DENV1, DENV2, DENV3, and DENV4, respectively, compared to each infection control. This decrease in titer displayed a relationship with the production of the NS1 viral protein, demonstrating inhibition of the viral genome. Although evaluation of siRNA may be conducted through various methods, liposome formulations are safer when seeking to perform future in vivo evaluations. Moreover, the immunological mechanisms through which the liposomes may be recognized would permit increased in vivo transfection efficiency [57,67]. Due to the nature of liposomes, lipid nanoparticles are considered safe for use in living organisms. The effectiveness of their safety depends on the nature of their components, their lipid organization, size, surface charge, and the molecules encapsulated in them, which can induce chemical modifications on the lipid particle that can result in improved effectiveness in the administration of drugs, peptides, or nucleic acids, however, this may affect the liposome’s safety, leading to toxigenic reactions [57,67,68]. With this in mind, a cytotoxic evaluation was conducted of erythrocyte lysis and cytokine release tests, which showed that the formulation carried out in this work produced no cytopathic effects, did not cause lysis, and did not stimulate release of pro-inflammatory cytokines in comparison with controls. These results, although obtained through an in vitro evaluation, agree with in vivo studies using formulations of liposomes with cationic lipids, which reinforces the theory in which liposomes carried out in this work could be used in live models [24,25,64,65]. Formulations by Cullis et al. [63] with cationic lipids for siRNA delivery for treatment against cancer have shown favorable results for therapy in live models. These formulations, unlike the liposomes from the present work, did not have poly(ethylene glycol)-lipid (PEG-lip), which supports the stability of liposomes in solutions, like plasma and allows the molecule to persist longer in the organism. Furthermore, PEG also permits the generation of smaller particles between 25 and 100 nm in diameter, providing observable differences given that with the lipid formulation from this work exhibited a mean size of 250 nm [69]. This component, although generating improvement on the lipid particles or liposomes, may also generate some allergic or immune reaction [24,69]. It is possible that the lack of a cellular reaction from the liposomes obtained herein is due to the lack of PEG-lip. It is then clear that PEG is a component to be considered in future in vivo trials [55,56,70]. Finally, liposomes with siRNA were evaluated against the four serotypes of dengue virus, which revealed antiviral activity, they did not cause cell damage (HepG2, human erythrocytes), and did not induce release of pro-inflammatory cytokines. Therefore, their evaluation in live models should be considered. On the other hand, there remains the possibility of evaluating these siRNAs mixed in pools for use in live models or to perform an evaluation on other flaviviruses given the genetic distance between these viruses.

## Figures and Tables

**Table 1:** Overall parameters of the siRNA obtained.

Name	Sequence	Target Protein Position	%GC	* BFE	** FBE	Predicted Inhibition Efficiency
DV1-siRNA 1.1	5′CACUGCAUGGGACUUUGGUUCUAUA 3′ 5′ UAUAGAACCAAAGUCCCAUGCAGUG 3′	E	44	2.1	−45.7	1.006
DV1-siRNA 1.2	5′ GAGCAUGGAACAUUUGGGAAGUUGA 3′ 5′ UCAACUUCCCAAAUGUUCCAUGCUC 3′	NS1	44	2.1	−45.1	1.014
DV1-siRNA 2.1	5′ CCUCCAUUUGGAGACAGCUACAUCA 3′ 5′ UGAUGUAGCUGUCUCCAAAUGGAGG 3′	E	48	−5.2	−48	0.947
DV1-siRNA 2.2	5′ CCUGGGAGGAGUGUUCACAUCUAUA 3′ 5′ UAUAGAUGUGAACACUCCUCCCAGG 3′	E	48	1.9	−48.4	1.021
DV1-siRNA 3.1	5′ CCUGGAACAAGAAAGAGCUUCUUGU 3′ 5′ ACAAGAAGCUCUUUCUUGUUCCAGG 3′	E	44	−5.0	−44.9	0.975
DV1-siRNA 3.2	5′ GAGCAAACAGGAGUGUCCCACAAUU 3′ 5′ AAUUGUGGGACACUCCUGUUUGCUC 3′	NS2B	48	−3.1	−46.3	1.004
DV1-siRNA 4.1	5′ CCACAUUCAGGAAUGGGAUUGGAAA 3′ 5′ UUUCCAAUCCCAUUCCUGAAUGUGG 3′	PreM	44	−2.5	−45.1	0.971
DV1-siRNA 4.2	5′ CAGAAGCAAAGAUGCUGCUUGACAA 3′ 5′ UUGUCAAGCAGCAUCUUUGCUUCUG 3′	NS3	44	−4.7	−44.8	0.975
DV1-siRNA 1	5′ UGUUAGAACUACGUUAAGCAA 3′ 5′ GCUUAACGUAGUUCUAACAGU 3′	C	36	1.7	−31.7	1.02
DV1-siRNA 2	5′ UGAUUAUGCAGCACAUUCCCA 3′ 5′ GGAAUGUGCUGCAUAAUCACG 3′	NS4A	45	−1.3	−37	0.996
DV1-siRNA 3	5′ UCUUUUCAACCCUUCUUUGGC 3′ 5′ CAAAGAAGGGUUGAAAAGAGG 3′	NS5	43	1.8	−35.7	0.94
DV1-siRNA 4	5′ UUUUUUGAGCUCUCUAUCCAA 3′ 5′ GGAUAGAGAGCUCAAAAAACC 3′	NS1	38	1.8	−32.2	1.019

* BFE: Bending free energy. ** FBE: Free binding energy (Kcal/mol).

## Supplementary Materials

The following are available online at https://www.mdpi.com/article/10.3390/v14020339/s1, Figure S1: Cell viability of the HepG2 cells at the end of each transfection assay from a. DENV1 b. DENV2 c. DENV3 d. DENV4. e. It shows the HepG2 morphology after transfection with siRNA (CN) and f. the effectiveness of transfection with siRNA (FITC Conjugate) like transfection positive control. Graphic and analysis constructed in GraphPad-Prism 9. Figure S2. a. The interaction of the erythrocytes with the four L-siRNAs did not generate hemoglobin release as a consequence of erythrocyte lysis (CC: cells control, HC: hemolysis control). b. Cell viability of the HepG2 cells at the end of each transfection assay and infection with DENV. Tukey’s multiple comparisons test * *p* < 0.05 ** *p* < 0.001 *** *p* < 0.0001 **** *p* < 0.00001. Graphic and analysis constructed in GraphPad-Prism 9.
