# Supplementary figures and images for: In Vitro Inhibition of Replication of Dengue Virus Serotypes 1–4 by siRNAs Bound to Non-Toxic Liposomes
Source: Viruses. 2022 Feb 7;14(2):339. doi: 10.3390/v14020339 (PMC8875542; doi:10.3390/v14020339)

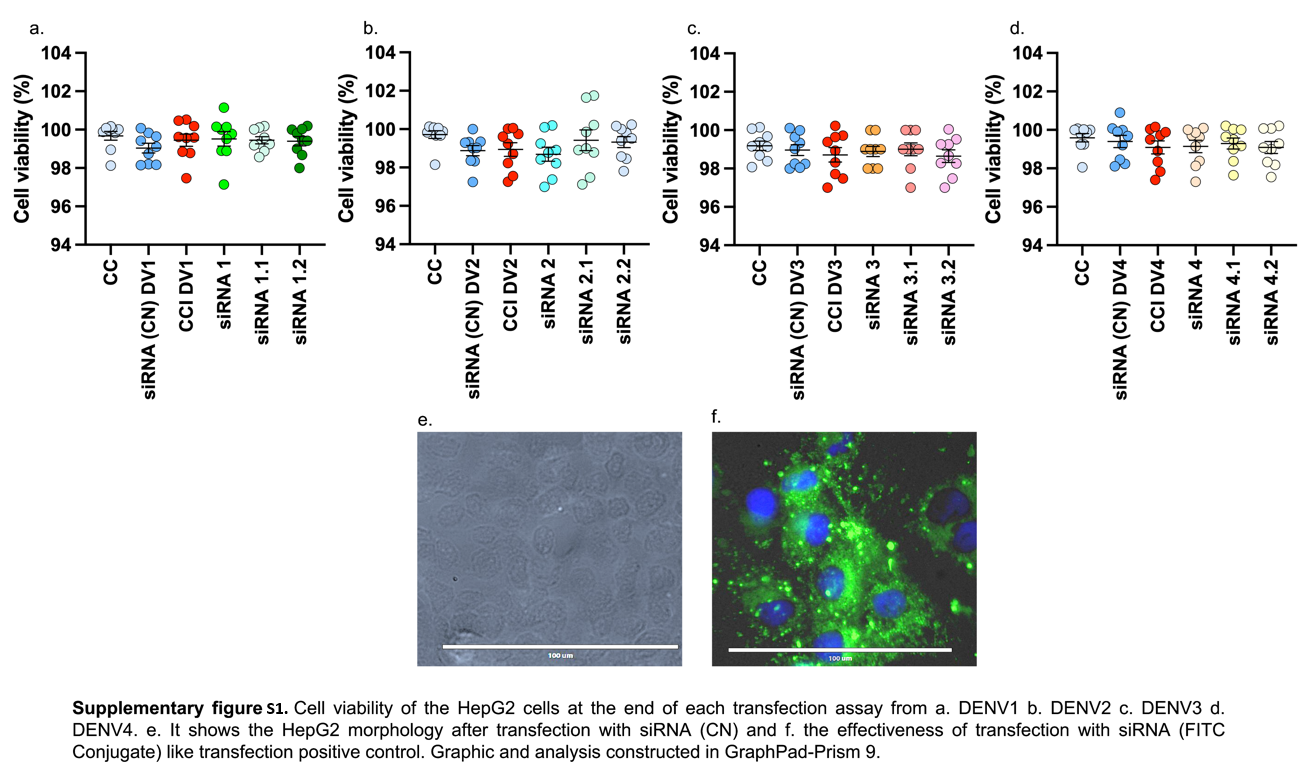

Supplement: Supplementary file 1 [file viruses-14-00339-s001.zip › supplementary figure S1.png]

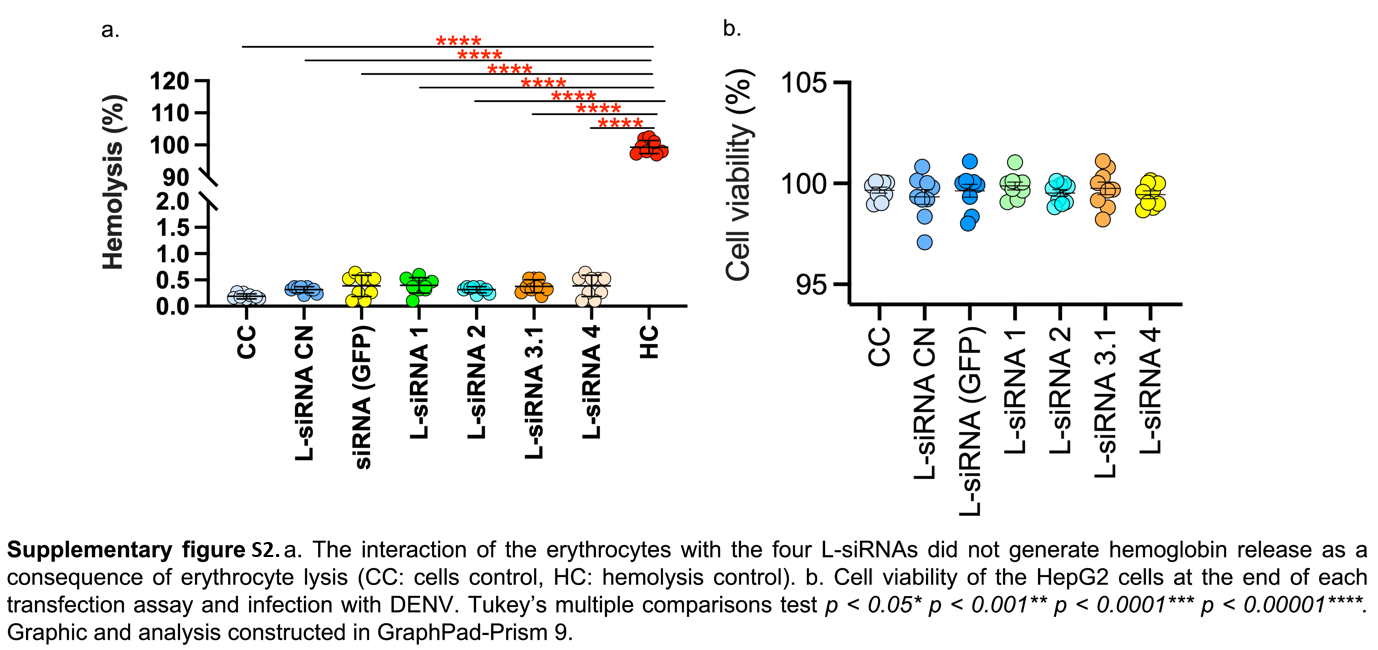

Supplement: Supplementary file 1 [file viruses-14-00339-s001.zip › supplementary figure S2.png]
